# Maternal High-Protein Diet during Pregnancy Modifies Rat Offspring Body Weight and Insulin Signalling but Not Macronutrient Preference in Adulthood

**DOI:** 10.3390/nu11010096

**Published:** 2019-01-05

**Authors:** Gabrielle Carlin, Catherine Chaumontet, François Blachier, Pierre Barbillon, Nicolas Darcel, Anne Blais, Corine Delteil, Florence M. Guillin, Sophie Blat, Eline M. van der Beek, Andrea Kodde, Daniel Tomé, Anne-Marie Davila

**Affiliations:** 1 UMR PNCA, AgroParisTech, INRA, Université Paris-Saclay, 75005 Paris, France; gabrielle.carlin@agroparistech.fr (G.C.); catherine.chaumontet@agroparistech.fr (C.C.); francois.blachier@agroparistech.fr (F.B.); nicolas.darcel@agroparistech.fr (N.D.); anne.blais@agroparistech.fr (A.B.); corine.delteil@agroparistech.fr (C.D.); florence.guillin@agroparistech.fr (F.M.G.); daniel.tome@agroparistech.fr (D.T.); 2 UMR MIA-Paris, AgroParisTech, INRA, Université Paris-Saclay, 75005 Paris, France; pierre.barbillon@agroparistech.fr; 3 INRA, INSERM, Univ Rennes, Nutrition Metabolisms and Cancer, NuMeCan, 35000 Rennes, France; sophie.blat@inra.fr; 4 Danone Nutricia Research, 3584 CT Utrecht, The Netherlands; eline.vanderbeek@danone.com (E.M.v.d.B.); andrea.kodde@danone.com (A.K.); 5 Dept Pediatrics, University Medical Centre Groningen, University of Groningen, 9713 GZ Groningen, The Netherlands

**Keywords:** high-protein diet, gestation, rat, food preferences, dietary self-selection, programming, adiposity

## Abstract

Diet of mothers during gestation may impact offspring phenotype. This study evaluated the consequences of a maternal High-Protein (HP) diet during gestation on food preferences and phenotypic characteristics in adult rat offspring. Dams were fed a HP or a Normal-Protein (NP) isocaloric diet during gestation only. Weaned female pups were divided into 3 diet groups: NP control or one of two dietary self-selection (DSS) conditions. In DSS1, offspring had a free choice between proteins (100%) or a mix of carbohydrates (88%) and lipids (12%). In DSS2, the choice was between proteins (100%), carbohydrate (100%) or lipids (100%). DSS2 groups consumed more of their energy from protein and lipids, with a decreased carbohydrate intake (*p* < 0.0001) compared to NP groups, regardless of the maternal diet. Offspring from HP gestation dams fed the DSS2 diet (HPDSS2) had a 41.2% increase of total adiposity compared to NPDSS2 (*p* < 0.03). Liver Insulin receptor and Insulin substrate receptor 1 expression was decreased in offspring from HP compared to NP gestation dams. These results showed the specific effects of DSS and maternal diet and data suggested that adult, female offspring exposed to a maternal HP diet during foetal life were more prone to adiposity development, in response to postweaning food conditions.

## 1. Introduction

During the perinatal period, foetus exposure to challenging environmental conditions and particularly inappropriate nutrition, is critical and generates programming effects often revealed in adulthood [1,2]. The foetus is particularly sensitive to the mother’s diet during pregnancy [3,4,5,6,7]. Studies show that the composition of the maternal diet may affect appetite and control of energy balance in the offspring [8,9,10] and that flavour exposure in utero represents a determinant for later food preferences [11]. These processes are instrumental for feeding behaviour and metabolic phenotype but may also influence the predisposition to metabolic diseases including type 2 diabetes and obesity [2,12].

Among the macronutrients of the maternal diet during pregnancy, protein quantity has been shown to modulate feeding behaviour and metabolic function in rat offspring [13]. A low-protein (LP) diet during gestation increases the risk of offspring being overweight and having food control disorders in adulthood [14,15,16,17]. Moreover, female offspring from LP dams, when put on dietary self-selection (DSS) and allowed to choose among various diet options, showed a preference for a high-fat diet at 12 weeks of age [18]. The impact of a maternal high-protein (HP) diet during gestation, though less thoroughly investigated, could impact glucose homeostasis [19,20] and increase adiposity [21,22] in rat offspring. Prospective studies in human groups indicate that higher maternal protein intake during pregnancy is positively associated with an increased body mass index in the offspring [23,24].

The aim of the present study was to determine the effects of a maternal HP diet during gestation on food preferences and metabolic risks in female adult offspring. Therefore, rat dams were exposed to either HP or Normal-Protein (NP) diet during gestation. Female offspring were then selected and were subsequently put on DSS for an 11 week-period. Females were selected because they showed a higher sensitivity to early foetal environment changes than males [20,25]. Data on energy intake, macronutrient choices, body weight and body composition were collected throughout the study; data on plasma glucose, metabolic hormones and gene expression in liver, adipose tissue and brain were assessed at the end of the study.

## 2. Materials and Methods

### 2.1. Experimental Design and Diets

**Figure 1:**
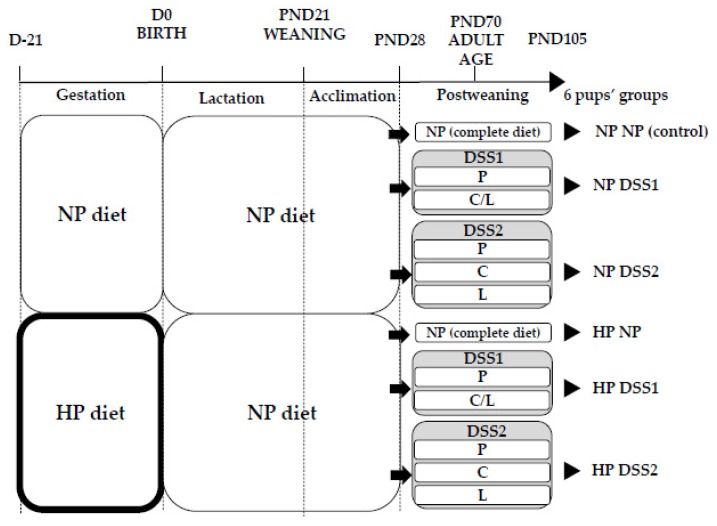
Six experimental groups formed. Dams received a NP control diet or a HP diet during gestation only. From PND28 until PND105, pups were divided into 3 groups from each dam group: NP diet (control, no free choices), DSS1 with P and G/L in 2 different cups and DSS2 with P, G and L in 3 different cups. D, Day; PND, Post-Natal Day; NP, Normal-Protein (control); HP, High-Protein; DSS, Dietary Self-Selection; P, Proteins; C/L, Carbohydrates and Lipids mix; C, Carbohydrates; L, Lipids.

Animal experiments were conducted according to the European legislation on animal experimentation and validated and approved by the Ethics Committee in Animal Experiment of INRA Jouy-en-Josas and French Research Minister (COMETHEA, registration number: APAFIS#3988-2016011910059852). Sixteen 6-week-old female and 6 male Wistar rats (HsdHan^®^:WIST, Envigo, France) were maintained under controlled housing conditions (22 ± 1 °C, 12 h-light/12 h-dark cycle with lights on at 8:00 am) with free access to food and water. After 1 week of habituation, dams were housed individually and mated for one week. From the day of mating, they were concomitantly randomized to two different isocaloric diets throughout gestation, a NP control (*n* = 7) or HP (*n* = 9) diet. The NP diet was composed of 20% energy from cow milk protein (Ingredia, France), 10% energy from fat and 70% energy from carbohydrates; the HP diet was composed of 55% energy from cow milk proteins, 10% energy from fat and 35% energy from carbohydrates (see Table 1). During lactation all dams were fed the NP diet. At birth, litters were standardized to 8 pups, prioritizing females. On postnatal day (PND) 21, pups were weaned and 48 female pups were selected and fed *ad libitum* with the NP diet. On PND28, female rat pups were randomly selected, individually housed and started on the self-selection protocol up to PND105. Pups selected in each group originated from 3 or 4 different litters. The offspring were subjected, *ad libitum*, to either the control NP diet (no choice) or one of the two DSS paradigms: the DSS1 (choice #1) diet or the DSS2 (choice #2) diet. The DSS1 group was given the choice between 2 cups with either protein (P) (100%) or a mix of carbohydrate (C) (88%) and lipid (L) (12%) [26]; the DSS2 group was given the choice between 3 cups, with either protein (P) (100%), carbohydrate (C) (100%) or lipid (L) (100%) [27] (Figure 1). Then, six experimental groups were formed: NPNP (*n* = 8), HPNP (*n* = 7), NPDSS1 (*n* = 8), HPDSS1 (*n* = 8), NPDSS2 (*n* = 8) and HPDSS2 (*n* = 8). All diets, including self-selecting diets, were based on the AIN-93G diet composition requirements for pregnant/lactating rats or growing rats [28] and were equally balanced in non-digestible fibres, vitamins and minerals (see diet composition details in Table 1). Offspring were weighed daily from birth to PND105 (see details on animal procedure in Supplementary Materials Methods S1). From PND28, food intake was monitored at 24 h intervals, except weekends, throughout the entire study. The total energy intake (in kcal) of each rat was determined from food weighing data and the known energy content of each diet. Since the exact macronutrient composition of each diet was known, the percentage of the total energy intake attributed to each macronutrient was calculated as well.

### 2.2. Body Composition Measurement by Magnetic Resonance Imaging (MRI)

Fat pads (total, visceral and subcutaneous) were measured by in vivo MRI (Platform Imagerie du Vivant, Paris-Descartes, France) on PND28, 70 and 105 (eve of euthanasia). Images were defined with a 4.7 T Bruker Biospec system (running Paravision 5, Bruker BioSpin GmbH, Ettlingen, Germany) using a Bruker 70 mm i.d. tunable quadrature RF resonator. The anaesthesia was performed with isoflurane in oxygen-supplementary air, breathing rate being monitored and body temperature being maintained at 36–38 °C. Images registered were segmented (by fuzzy c-means) with MIPAV 7.3.0 (national institutes of health, Bethesda, MD, USA). Volume of adipose tissues was determined by quantification of the voxel’s mask directly converted to mm^3^ (see the example in Supplementary Materials Methods F1) and then to grams using a density value of 0.92 kg/dm^3^.

### 2.3. Sampling

On PND105 after overnight fasting, a first blood sample (~500 µL) was taken by tail vein puncture. Then animals were fed a calibrated meal (3 g of dry matter) during light phase, composed according to the previous week of each one’s macronutrient intake when included in a DSS group. The calibrated meal was removed after 30 min and over the following 60 min rats were anesthetized with an intraperitoneal injection of pentobarbital sodium before decapitation, (time + 90 min). A second blood sample was collected during dissection (fed state). The peri-ovarian adipose tissue, liver, pancreas, hypothalamus and isolated Nucleus Accumbens (NAcc) [29] were sampled, snap-frozen in liquid nitrogen and stored at −80 °C. Body composition was assessed by weighing the carcass, adipose tissues (subcutaneous, peri-ovarian, retroperitoneal and mesenteric) and other organs. Blood was centrifuged (3000 g, 4 °C, 15 min) and plasma was directly stored at −80 °C.

### 2.4. Biochemical Assays

Fasting blood glucose was immediately measured using a glucose meter (Accu-Check Go, Roche Diagnostic, Mannheim, Germany). Plasma glucose (fed state only), triglycerides (TG), protein and total cholesterol levels were determined using an Olympus AU400 automatic chemical analyser (Biochemistry and metabolism platform, Bichat Hospital, CRI, France). Plasma insulin was determined with ELISA immunoassay (Insulin ELISA, Mercodia, Uppsala, Sweden). The pancreatic content of insulin (500 mg of pancreas) was extracted in an acid- ethanol solution (HCl, ethanol, H20) and measured by fluoro-immunoassay (Insulin FIA, Mercodia, Uppsala, Sweden). The plasma levels of leptin, glucagon like peptide 1 (GLP1), peptide YY (PYY) and gastric inhibitory polypeptides (GIP) were measured with a Luminex assay (RMHMAG-84K-05, Rat Metabolic Hormone Magnetic Bead Multiplex Assay, Merck-Millipore, Billerica, MA, USA).

### 2.5. Gene Expression by Quantitative PCR

Total RNA from hypothalamus, NAcc, peri-ovarian adipose tissue (100 mg) and liver (100 mg) were extracted using 1 mL Trizol Reagent (Invitrogen, Carlsbad, CA, USA) and synthesis of cDNA was performed, as previously described [30], with 400 µg total RNA. Real-time PCR was performed on a StepOnePlus^TM^ real-time PCR (Applied Biosystems, Foster City, CA, USA) using SYBR green fast reagent PCR master mix (Applied Biosystems) under the following conditions: 10 min at 95 °C, 40 cycles of 95 °C for 15 s and 1 min at 60 °C. Primers sequences used are detailed in Supplementary Materials Table S1. Gene expression was determined using the formula below. Results of this gene expression are presented as an arbitrary unit using the NPNP group as reference sample (NPNP gene expression = 1).

2^−ΔΔ*C*t^ with ΔΔ*C*t = (*C*t**_sample_** gene of interest − *C*t**_sample_** 18S) − (*C*t**_reference sample_** gene of interest − *C*t**_reference sample_** 18S)

### 2.6. Statistical Analysis

Data on birth weight. One-way analysis of variance (ANOVA) was performed to determine the effect of the maternal diet during gestation (gestation factor).

Data after weaning. Two-way ANOVA (model W) was performed to determine the effect of the maternal diet during gestation (gestation factor), the postweaning diet (postweaning factor) and the interaction between both (gestation × postweaning). For repeated measurements(model Wt), a ‘time factor’ was added as a repeated factor and a ‘pup factor’ was added as a random factor to take into account that data were collected at differing time points on the same pup. In the two postweaning models (W and Wt), a random factor was added to correct for possible correlations between pups from the same litter.

Pairwise comparisons were adjusted with multiple comparisons using a Tukey post-hoc test. The statistical analyses were performed using R studio 1.1.456 (Boston, MA, USA) and the differences between groups were considered significant at *p* < 0.05. All data are expressed as means ± SEMs (Standard Error of the Mean).

## 3. Results

### 3.1. Offspring Energy Intake and Macronutrient Self-Selection

**Figure 2:**
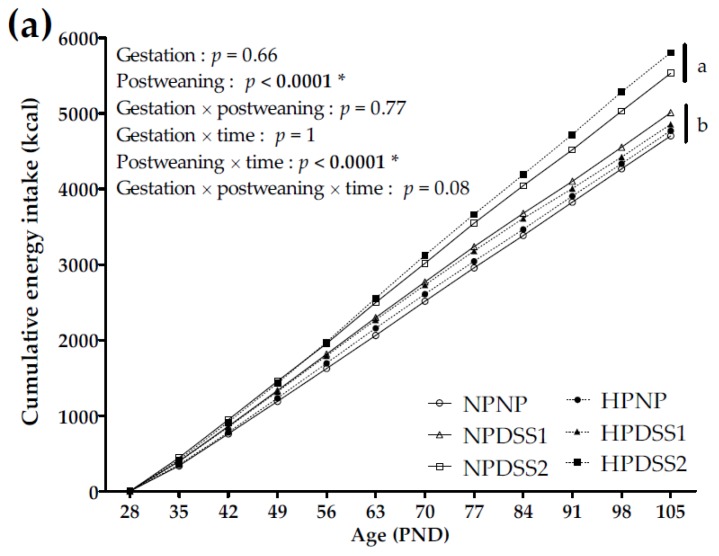

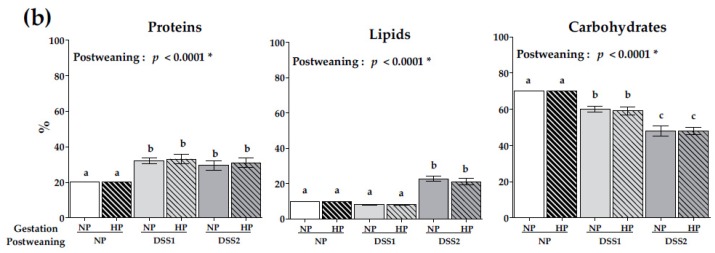
Cumulative energy intake during self-selection period from PND28 to PND105 (**a**) and macronutrient (Proteins, Lipids and Carbohydrates) consumption levels (% of total energy intake) on PND105 (**b**). Data are means ± SEMs. Effects of diets were tested within model W and Wt (*, *p* < 0.05). Means that are significantly different (*p* < 0.05) according to the post-hoc test have different letters (^a^ or ^b^ or ^c^). PND, Post-natal Day; NP, Normal-Protein (control); HP, High-Protein; DSS1, Dietary Self-Selection 1 (P and G/L in 2 different cups); DSS2, Dietary Self-Selection 2 (P, G and L in 3 different cups).

The cumulative energy intake (energy intake summed day by day) of the offspring fed either the NP or DSS diets (PND28 to PND105) is reported in Figure 2a. Macronutrient self-selection during the DSS post-weaning period of pups is reported in Figure 2b.

The maternal HP diet, compared to NP diet, did not affect cumulative energy intake (Figure 2a) or the contribution of macronutrients (Figure 2b) to the overall intake in offspring.

The postweaning diet significantly affected the cumulative energy intake (Figure 2a) and final energy intake (energy intake from PND28 to 105)—both were higher in the DSS2 groups compared to NP and DSS1 groups (NPNP, 4703.9 ± 78.3 kcal; HPNP, 4771.7 ± 103.6 kcal; NPDSS1, 5010.3 ± 101.3 kcal; HPDSS1, 4855.5 ± 163.3 kcal; NPDSS2, 5532.7 ± 226.2 kcal; HPDSS2, 5804 ± 176.7 kcal; postweaning, *p* < 0.0001; postweaning × time, *p* < 0.0001). Compared to the postweaning NP control groups, protein consumption was consistently and significantly higher in the DSS groups (postweaning, *p* < 0.0001) representing approximately 30% of the total energy intake. Carbohydrate consumption was significantly lower in the DSS groups, particularly in the DSS2 groups (postweaning, *p* < 0.0001). Lipid consumption was significantly higher in the DSS2 groups representing 22% of the total energy intake (postweaning, *p* < 0.0001). In summary, compared to the postweaning NP control groups on a low-fat/high-carbohydrate diet, the DSS1 groups increased protein intake and the DSS2 groups increased energy, protein and fat intakes (Figure 2b).

### 3.2. Offspring Body Weight Gain and Adiposity Development

**Figure 3:**
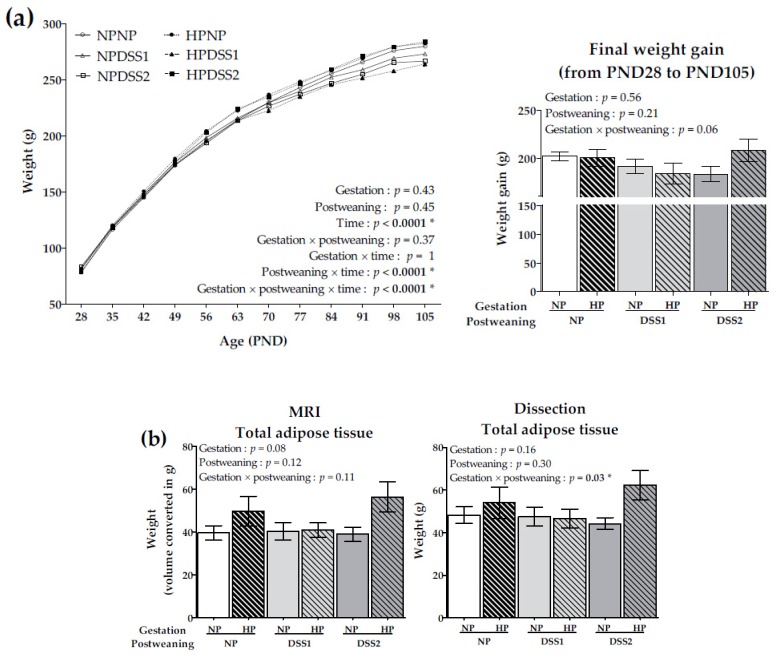
Body weight during the self-selection period from PND28 to PND105 and final weight gain on PND105 (**a**) and total adipose tissue weights on PND105 estimated by Magnetic Resonance Imaging (MRI) and by dissection (**b**). Data are means ± SEMs. Effects of diets were tested within model W and Wt (*, *p* < 0.05). PND, Post-natal Day; NP, Normal-Protein (control); HP, High-Protein; DSS1, Dietary Self-Selection 1 (P and G/L in 2 different cups); DSS2, Dietary Self-Selection 2 (P, G and L in 3 different cups); MRI: Magnetic Resonance Imaging.

Body weight between PND28 and PND105 and final weight gain are reported in Figure 3a. Weights (in g) of visceral (VAT) and subcutaneous (SAT) adipose tissues determined by MRI, on PND28, 70 and 105 are reported in Table 2. Total adipose tissue (TAT) weights (in g) determined by MRI on PND28 and 70 are also reported in Table 2. TAT weights determined by MRI and dissection on PND105 are presented in Figure 3b. Results from both MRI and dissection for final TAT were in accordance, although MRI results indicated significant differences (*p* < 0.0001) with approximately 15% lower final weights of TAT in each animal group.

Birth weight of pups was comparable between HP and NP gestation groups (NP, 5.38 ± 0.17 g; HP, 5.50 ± 0.17 g). During lactation, growth and pup weight were not affected by the gestation diet (NP, 47.8 ± 1.5 g; HP, 47.8 ± 0.8 g). On PND28, all pups (randomly distributed in groups) had a similar body weight (NPNP, 78.2 ± 3.4 g; HPNP, 80.3 ± 2.3 g; NPDSS1, 81.4 ± 3.8 g; HPDSS1, 80.4 ± 2.9 g; NPDSS2, 83.2 ± 2.9 g; HPDSS2, 79.5 ± 2.8 g). The gestation diet had no effect on the offspring body weight (Figure 3a). Adipose tissue weights were not significantly different between groups on PND28 (Table 2).

The postweaning diet over time significantly affected body weight (postweaning × time, *p* <0.0001) evidenced mainly by a lower body weight in the DSS1 groups (Figure 3a). On PND70, SAT was significantly increased in the DSS2 groups (*p* < 0.003) evidenced mainly by a higher increase in the HPDSS2 group (Table 2).

The gestation diet, in conjunction with the postweaning diet and time, affected body weight and displayed a significant effect mainly driven by a difference between NPDSS2 and HPDSS2 groups (gestation × postweaning × time, *p* < 0.0001) (Figure 3a). On PND105, the final VAT was significantly higher in the HPDSS2 group compared to NPDSS2 group (gestation × postweaning diet, *p* ≤ 0.02) regardless of the measurement method (Table 2).

### 3.3. Maternal HP Diet during Gestation Affects Fasting Leptin Levels

**Figure 4:**
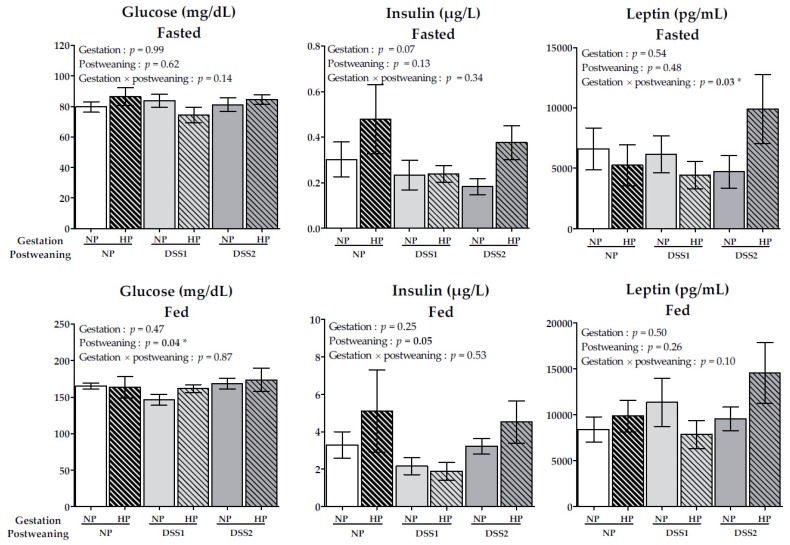
Fasted and fed (*90 minutes after calibrated meal)* blood and plasma glucose (mg/dL), insulin (µg/L) and leptin (pg/mL) levels. Data are means ± SEMs. Effects of diets were tested within model W (*, *P* < 0.05). NP, Normal-Protein (control); HP, High-Protein; DSS1, Dietary Self-Selection 1 (P and G/L in 2 different cups); DSS2, Dietary Self-Selection 2 (P, G and L in 3 different cups).

Blood, plasma glucose, insulin and leptin levels, from both fasting and fed states, are reported in Figure 4. Other blood and plasma parameters are reported in Table 3.

Pancreatic insulin levels (µg/L/g) in tissue on PND105 were comparable between groups (NPNP, 71.5 ± 15.2; HPNP, 96.0 ± 15.0; NPDSS1, 75.0 ± 19.1; HPDSS1, 62.6 ± 9.2; NPDSS2, 69.7 ± 17.9; HPDSS2, 67.4 ± 13.3). PYY, GLP1 and GIP plasma concentrations were relatively unaffected by diets (Supplementary Materials Table S2).

Blood and plasma parameters presented in Figure 4 and in Table 3 were not affected by the maternal diet during gestation.

Regarding postweaning feeding effects, plasma glucose levels in the fed state were higher in the DSS2 groups and lower in the DSS1 groups, compared to control groups (*p* < 0.05). Fed plasma insulin was lower, by just by enough to be considered significant (*p* = 0.05), in the DSS1 groups compared to the other postweaning groups (Figure 4). Fasted plasma TG levels were significantly decreased in both DSS groups, especially in the DSS2 groups (*p* < 0.05). However, no effects in fed TG plasma concentrations were found. The fed plasma cholesterol level was decreased in the DSS1 and DSS2 groups, compared to the control group (*p* < 0.05). Protein levels were not affected during fasting but decreased in fed plasma in the DSS1 and DSS2 groups (*p* < 0.01) (Table 3).

Unlike the fed plasma, fasting plasma concentrations of leptin on PND105 were significantly affected by the interaction between the gestation and postweaning diets (*P* < 0.05) (Figure 4).

### 3.4. Maternal HP Diet during Gestation Impacts Liver Insulin Signalling But Not Central Markers of Food Intake Regulation

**Figure 5:**
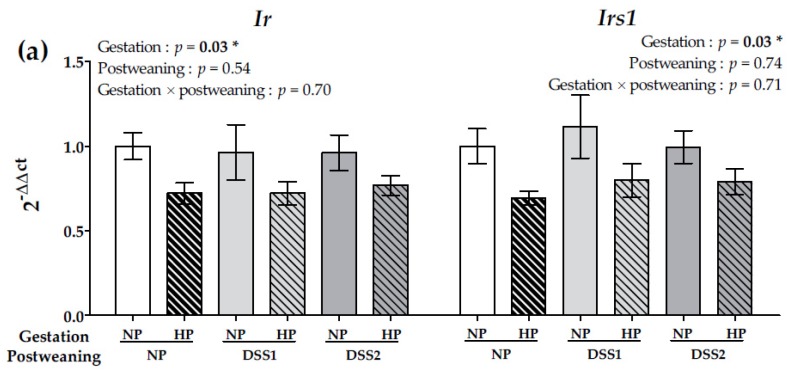

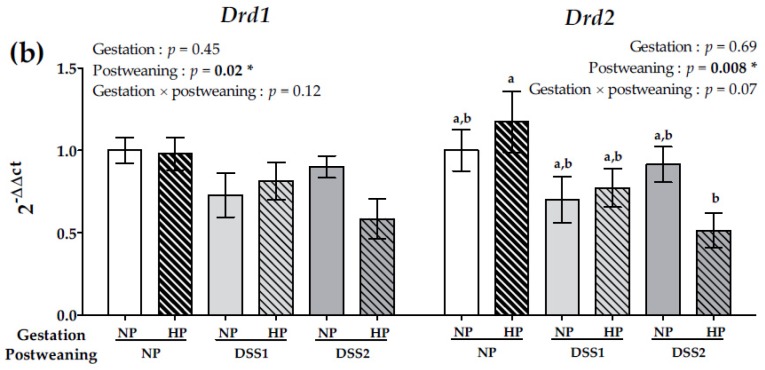
Gene expression in NAcc of Drd1 and Drd2 (**a**) and gene expression in liver of IR and IRS1 (**b**) on data collected on PND105. Data are means ± SEMs. Effects of diets were tested within model W (*, *p* < 0.05). Means that are significantly different (*p* < 0.05) according to the post-hoc test have different letters (^a^ or ^b^). Drd1 and *2*, Dopamine receptors 1 and 2; IR, Insulin receptor; IRS1, Insulin receptor substrate 1; PND, Post-natal Day; NP, Normal-Protein (control); HP, High-Protein; DSS1, Dietary Self-Selection 1 (P and G/L in 2 different cups); DSS2, Dietary Self-Selection 2 (P, G and L in 3 different cups).

In the liver, insulin receptor (*Ir*) and insulin receptor substrate (*Irs1*) expression significantly decreased with the HP maternal diet (*p* < 0.05) (Figure 5a).

Regarding postweaning diet effects, in the liver (Table 4), expression of glucokinase (*Gck*) significantly decreased in the DSS1 and notably in DSS2 groups (*p* < 0.05). Expression of fatty acid synthase (*Fas*), stearoyl-CoA desaturase 1 (*Scd1*) (*p* < 0.01) and sterol regulatory element-binding transcription factor 1 isoform c (*Screbf1c*) (*p* < 0.01) significantly decreased in DSS2 animals (*p* < 0.05). Phosphoenolpyruvate carboxykiase (*Pepck*) expression increased in the DSS1 and in DSS2 groups (*p* < 0.0001), mainly driven by a higher increase in HPDSS2 group. In adipose tissue, expression of Lipoprotein lipase (*Lpl*), *Fas*, *Acc*, *Scd1* and *Screbf1c*, patatin like phospholipase domain containing 2 (*Pnpla2*), leptin (*Lep*), peroxisome proliferator-activated receptor *gamma* (*Pparg*) was not affected by the diets (data not shown). In the hypothalamus, expression of neuropeptide Y (*Npy*) agouti-related protein (*Agrp*), pro-opiomelanocortin (*Pomc*), cocaine- and amphetamine-regulated transcript (*Cartpt*), leptin receptor (*Lepr*) and *Ir* was not changed by the diets (Supplementary Materials Table S3). In contrast, in NAcc, expression of both the dopamine receptor 1 (*Drd1*) (*p* = 0.02) and dopamine receptor 2 (*Drd2*) (*p* = 0.008) (Figure 5b) significantly decreased in rats put on DSS.

## 4. Discussion

The present study evaluated the influence of maternal HP diet during gestation on feeding behaviour using postweaning DSS feeding and on metabolic phenotype characteristics in adult female offspring. In fact, only few studies highlighted the specific metabolic mechanisms affected by maternal HP diet during gestation on adult offspring. The present work furthers the literature with a novel approach combining maternal protein diet modification and offspring food choices, through DSS, during growth and early adulthood. DSS mimics free choice in feeding by increasing available food options. The present analyses cover a wide range of phenotypic characteristics in an attempt to elucidate the links between maternal diet and food intake regulation and energy metabolism disorders in offspring. First, the results indicated that offspring put on DSS with separate macronutrient options preferentially consumed more lipids and less carbohydrates, irrespective of maternal feeding during gestation. In accordance with these specific food choices made by rats, the DSS1 diet can be considered as a high-protein/low-fat diet and the DSS2 diet as a high-protein/high-fat diet. Second, the HP maternal diet (vs. NP) altered insulin signalling in the liver of offspring. Third, the HP maternal diet (vs. NP) induced higher adiposity in offspring fed either the DSS2 diet or (to a lesser degree) the high-carbohydrate NP diet but not those fed the DSS1 diet.

Birth weight was not affected by the gestation HP diet, as shown in a previous work [20]. The impact of the gestation HP diet on weight gain was most prominent under both the DSS2 condition and, to a lesser extent, the postweaning high-carbohydrate NP diet. Weight gain was higher throughout the study in HPDSS2 compared to NPDSS2 rats, although energy intake was not significantly different between the two. The higher weight gain in HPDSS2 rats was paralleled by increased fat storage and a two fold increase of the fasting plasma leptin level compared to NPDSS2 rats. Accordingly, total body adiposity and circulating leptin levels were affected by the maternal diet during gestation, even though gene expression in adipose tissue was not affected. Adipose tissue is a key endocrine-like organ secreting leptin in proportion to adipose tissue mass. Leptin acts as a signal of long-term energy balance providing feedback to central energy regulation by steering the balance between energy consumption and expenditure [31]. The results showed that, compared to the postweaning NP control diet, the DSS either offered as DSS1 or DSS2 diets led to a higher protein intake of around 30% of the total energy intake, as previously observed after weaning or in adulthood [26,27,32,33]. However, the increase of fat intake at the expense of carbohydrates observed in DSS2 rats was associated with higher energy intake, compared to both NP and DSS1 rats. Although foetal exposure to HP diet was not studied in association to postweaning DSS, it was reported that LP diet during gestation could lead to a preference for fatty foods in 12-week-old female offspring [18]. Hyperphagia, as observed in the DSS2 condition, may be promoted when nutrients are given separately [34,35] or offered as a palatable diet [36]. It could be hypothesized that both NP and DSS2 diets, characterized by high-carbohydrate or high-fat contents, respectively, simulated an obesogenic diet, revealing an increased risk of being overweight as a result of HP foetal programming.

In this study, insulin levels in the fed state did not show any effect of the maternal diet, whereas fasting plasma insulin increased slightly in offspring from the HP maternal group and especially in the HPDSS2 and HPNP groups. Insulin is a key hormone regulating glycemia and nutrient storage. Deregulation of these metabolic processes can signal metabolic disorders [37] and a basal hyperinsulinemic state that can cause insulin resistance [38]. Interestingly, the present study showed that the maternal HP diet induced decreased *Insr* and *Irs1* expression in the offspring liver regardless of postweaning diet. It has been shown that a HP diet during gestation can induce differences in offspring liver transcriptome [39] and modified gene expression of key metabolism genes in the liver [22]. Because the liver is the major site of insulin clearance, dysfunctional insulin capture for degradation is a risk factor for type 2 diabetes and obesity [40]. Inactivation of *Ir* can induce hyperinsulinemia, reduce insulin clearance and impair carbohydrate homeostasis in liver-specific *Ir* knockout mice [41]. In addition, hepatic expression of *Pepck* expression was increased particularly in the HPDSS2 group indicating a higher hepatic glyconeogenesis compared to the NPDSS2 group. Changes in insulin function, for instance through a decrease in receptor expression, could explain overexpression of *Pepck* in the HPDSS2 group [42]. As in the present work, previous studies showed that a HP diet during gestation was associated with an increase of adiposity in rat offspring [20,21]. The latter is most likely a direct consequence of a foetal nutritional adjustment to the HP and low-carbohydrate environment, fostering increased gluconeogenesis [43]. Moreover, insulin is also a signal of long-term energy balance [44,45]. This finding highlighted that a gestation HP diet had a potential programming effect on offspring liver development that may impact body physiology differently, depending on the postweaning dietary environment.

In the hypothalamic arcuatus nucleus, leptin activates POMC and CARTPT producing neurons and inhibits NPY and AGRP [46]. In the present study, however, no effect was observed on the expression of *Pomc*, *Cartpt*, *Npy*, *Agrp* on PND105, despite the fact that leptin levels were normally higher in the HP gestation groups, particularly in HPDSS2 rats. Leptin is known to be higher in obese subjects [47] and obesity can promote dysregulation of leptin signalling leading to central leptin resistance [48,49]. The dopamine system plays a central role in reward and in food preference [50]. NAcc dopamine receptor measurements showed that *Drd1* and *Drd2* expression was significantly decreased by DSS. DRD2 is well known in human and animal models as an actor favouring obesity [51]. The DSS2 groups had a higher intake of lipids, which is in line with previous studies showing that long-term exposure to high-fat food results in downregulation of *Drd2* expression in reward centres [52,53,54]. It is tempting to speculate that there is a link between the lipid intake and observed DRD2 changes in our study—but that its significance is unknown. Moreover, these results showed that free-choice models (DSS) played a key role in hedonic food perception and that the central dopamine pathway was not modified by maternal HP diet during gestation. Nevertheless, studies in rodents focused on a maternal high-fat-high-sugar (HFHS) diet during early development, including gestation, showed that a HFHS diet increased the preference for palatable foods through alterations in the central dopamine circuit and a decrease of *Drd2* expression in NAcc [55] or in the ventral tegmental area [56].

## 5. Conclusions

In conclusion, the present study showed that, in rats, a maternal HP diet during gestation followed by a postweaning DSS2 feeding, composed of three separate macronutrients, increased the susceptibility to energy balance disorders and obesity in female offspring. In the case of the DSS condition, the dopamine circuit was affected through the decrease of *Drd1* and *Drd2*, suggesting that long-term DSS exposure could impact the hedonic food circuit. Offspring macronutrient choices were affected by the way the food was proposed; when the macronutrients were kept separate from each other, pups consumed more proteins and lipids and less carbohydrates, compared to control groups with a complete diet (NP). In addition, the maternal HP diet during gestation induced a decrease of *Ir* and *Irs1* expression in offspring livers, suggesting programming effects on the insulin signalling that may underlie the probable alterations in energy homeostasis. The effects of maternal LP diet [57,58] and maternal HP diet as showed in the present study, suggest the existence of an optimal protein/energy ratio in maternal diet during gestation for offspring health risk. These observations confirm the major role of maternal perinatal nutrition and showed the protein level during gestation as a determinant for phenotype programming with latter consequences on development of metabolic risks.

## Figures and Tables

**Table 1:** Diet composition.

	NP	HP	P	C/L	C	L
Composition						
Metabolizable energy (kcal/g)	3.8	3.8	3.6	3.9	3.6	8.1
Proteins (% energy)	20	55	100	-	-	-
Carbohydrates (% energy)	70	35	-	88	100	-
Lipids (% energy)	10	10	-	12	-	100
Ingredients (g/kg)						
Cow milk protein	200	530	902.7	-	-	-
Corn starch	570	287	-	732.3	776.4	-
Sucrose	92.7	45.7	-	119.1	126.3	-
Soybean oil	40	40	-	51.3	-	902.7
Mineral mix (AIN-93-MX)	35	35	35	35	35	35
Vitamin mix (AIN-93-VX)	10	10	10	10	10	10
Cellulose	50	50	50	50	50	50
Choline	2.3	2.3	2.3	2.3	2.3	2.3
Ratios (g/g)						
Carbohydrates/lipids	16.6	8.3	-	16.6	-	-
Corn starch/sucrose	6.2	6.3	-	6.2	6.2	-

NP, Normal-Protein (control); HP, High-Protein; P, Proteins; C/L, Carbohydrate and Lipid mix; C, Carbohydrates; L, Lipids.

**Table 2:** Adipose tissue (total, visceral and subcutaneous) weights (g) on PND28, 70 and 105.

Gestation Diet		NP			HP			*P*		
Postweaning Diet		NP (*n* = 8)	DSS1 (*n* = 8)	DSS2 (*n* = 8)	NP (*n* = 7)	DSS1 (*n* = 8)	DSS2 (*n* = 8)	Gestation	Postweaning	Gestation × Postweaning
PND28										
MRI	TAT	6.5 ± 0.4	6.5 ± 0.5	6.7 ± 0.5	6.9 ± 0.3	7.2 ± 0.3	7.0 ± 0.5	0.21	0.80	0.39
	VAT	4.5 ± 0.3	4.5 ± 0.3	4.7 ± 0.4	4.8 ± 0.3	5.1 ± 0.2	4.9 ± 0.4	0.34	0.96	0.66
	SAT	2.0 ± 0.2	2.0 ± 0.2	2.0 ± 0.2	2.1 ± 0.1	2.1 ± 0.1	2.1 ± 0.2	0.23	0.75	0.31
PND70										
MRI	TAT	27.3 ± 2.5	28.3 ± 2.5	26.5 ± 2.9	33.5 ± 5.2	30.4 ± 1.9	36.7 ± 4.5	0.06	0.34	<0.10
	VAT	19.4 ± 1.9	19.0 ± 2.2	16.7 ± 1.9	23.2 ± 2.5	19.9 ± 1.7	23.7 ± 2.4	0.07	0.46	<0.10
	SAT	7.9 ± 0.7 ^a,b^	9.4 ± 0.7 ^a,b^	10.2 ± 1.1 ^a,b^	10.3 ± 3.3 ^a^	10.6 ± 0.8 ^a,b^	15.0 ± 2.5 ^b^	0.18	0.003 *	0.25
PND105										
MRI	VAT	30.1 ± 2.5 ^a,b^	29.4 ± 3.2 ^a,b^	27.8 ± 2.3 ^a,b^	35.0 ± 3.9 ^a,b^	29.6 ± 3.1 ^a^	40.8 ± 4.8 ^b^	0.09	0.09	0.02 *
	SAT	9.4 ± 1.1	10.9 ± 1.1	11.1 ± 1.3	14.6 ± 3.4	11.3 ± 0.6	15.6 ± 2.5	<0.10	0.32	0.70
Dissection	VAT	29.0 ± 2.3 ^a,b^	28.2 ± 3.0 ^a,b^	26.0 ± 1.9 ^a,b^	33.3 ± 4.5 ^a,b^	28.5 ± 3.1 ^a^	39.4 ± 4.5 ^b^	0.09	0.18	0.008 *
	SAT	18.6 ± 1.6	18.4 ± 1.4	17.5 ± 1.0	20.0 ± 3.0	17.3 ± 1.6	22.2 ± 2.5	0.37	0.54	0.24

Data are means ± SEMs. Effects of diets were tested within model W (*, *p* < 0.05). Means that are significantly different (*p* < 0.05) according to the post-hoc test have different letters (^a^ or ^b^). TAT, Total Adipose Tissue; VAT, Visceral Adipose Tissue; SAT, Subcutaneous Adipose Tissue; MRI, Magnetic Resonance Imaging; PND, Post-natal Day; NP, Normal-Protein (control); HP, High-Protein; DSS1, Dietary Self-Selection 1 (P and G/L in 2 different cups); DSS2, Dietary Self-Selection 2 (P, G and L in 3 different cups).

**Table 3:** Metabolites in plasma on PND105.

Gestation Diet		NP			HP			*P*		
Postweaning Diet		NP (*n* = 8)	DSS1 (*n* = 8)	DSS2 (*n* = 8)	NP (*n* = 7)	DSS1 (*n* = 8)	DSS2 (*n* = 8)	Gestation	Post-weaning	Gestation × postweaning
Triglycerides (mmol/L)	Fasted	0.81 ± 0.13	0.72 ± 0.18	0.54 ± 0.06	0.71 ± 0.07	0.64 ± 0.11	0.48 ± 0.09	0.64	0.03 *	0.91
	fed	1.14 ± 0.14	1.17 ± 0.22	1.16 ± 0.22	1.81 ± 0.38	1.23 ± 0.19	1.18 ± 0.19	0.30	0.34	0.19
Cholesterol (mmol/L)	Fasted	1.19 ± 0.09	1.11 ± 0.09	1.19 ± 0.13	1.12 ± 0.15	0.87 ± 0.04	1.09 ± 0.09	0.10	0.19	0.68
	fed	1.74 ± 0.11	1.49 ± 0.13	1.65 ± 0.09	1.82 ± 0.23	1.40 ± 0.09	1.54 ± 0.10	0.67	0.04 *	0.72
Proteins (g/L)	Fasted	59.7 ± 1.8	58.2 ± 2.6	56.8 ± 1.5	57.5 ± 3.8	53.2 ± 1.6	54.7 ± 1.2	0.08	0.32	0.74
	fed	58.5 ± 1.4 ^a^^,^^b^	53.2 ± 3.3 ^a^	57.3 ± 0.8 ^a^^,^^b^	61.6 ± 1.3 ^b^	56.1 ± 1.0 ^a^^,^^b^	55.4 ± 1.1 ^a^^,^^b^	0.36	0.009 *	0.27

Data are means ± SEMs. Effects of diets were tested within model W (*, *p* < 0.05). Means that are significantly different (*p* < 0.05) according to the post-hoc test have different letters (^a^ or ^b^). PND, Post-natal Day; NP, Normal-Protein (control); HP, High-Protein; DSS1, Dietary Self-Selection 1 (P and G/L in 2 different cups); DSS2, Dietary Self-Selection 2 (P, G and L in 3 different cups).

**Table 4:** Gene expression in liver on PND105.

Gestation Diet	NP			HP			*P*		
Postweaning Diet	NP (*n* = 8)	DSS1 (*n* = 8)	DSS2 (*n* = 8)	NP (*n* = 7)	DSS1 (*n* = 8)	DSS2 (*n* = 8)	Gestation	Postweaning	Gestation × Postweaning
*Gck*	1.00 ± 0.20	0.79 ± 0.19	0.60 ± 0.10	0.91 ± 0.29	0.62 ± 0.19	0.30 ± 0.06	0.20	0.02 *	0.86
*Pklr*	1.00 ± 0.13	0.90 ± 0.10	0.75 ± 0.04	0.88 ± 0.09	0.71 ± 0.10	0.73 ± 0.06	0.20	0.06	0.62
*Fas*	1.00 ± 0.24	0.95 ± 0.14	0.58 ± 0.07	1.01 ± 0.17	0.77 ± 0.15	0.64 ± 0.09	0.76	0.03 *	0.69
*Acc*	1.00 ± 0.22	0.91 ± 0.17	0.65 ± 0.10	0.73 ± 0.10	0.69 ± 0.10	0.61 ± 0.08	0.16	0.17	0.66
*Scd1*	1.00 ± 0.25	0.88 ± 0.10	0.40 ± 0.12	0.62 ± 0.13	0.75 ± 0.22	0.43 ± 0.15	0.45	0.002 *	0.62
*Screbf1c*	1.00 ± 0.13	1.14 ± 0.21	0.710 ± 0.18	0.96 ± 0.14	0.78 ± 0.10	0.61 ± 0.09	0.29	0.009 *	0.52
*Pepck*	1.00 ± 0.12 ^a^	1.74 ± 0.44 ^a^	2.26 ± 0.19 ^a,b^	1.70 ± 0.80 ^a^	2.29 ± 0.66 ^a,b^	4.58 ± 0.77 ^b^	0.09	<0.0001 *	0.09

Data are means ± SEMs. Effects of diets were tested within model W (*, *p* < 0.05). Means that are significantly different (*p* < 0.05) according to the post-hoc test have different letters (^a^ or ^b^). GCK, Glucokinase; PKLR, Pyruvate kinase L/R; FAS, Fatty acid synthase; ACC, Acetyl-CoA carboxylase; SCD1, Stearoyl-CoA desaturase 1; SREBF1C, Sterol regulatory element-binding transcription factor 1 isoform c; PEPCK, Phosphoenolpyruvate carboxykinase; PND, Post-natal Day; NP, Normal-Protein (control); HP, High-Protein; DSS1, Dietary Self-Selection 1 (P and G/L in 2 different cups); DSS2, Dietary Self-Selection 2 (P, G and L in 3 different cups).

## Supplementary Materials

The following are available online at http://www.mdpi.com/2072-6643/11/1/96/s1, Methods S1: Animal procedure details during each experimental period, Methods F1: Example, for each diet group, of voxel masks of total (a), subcutaneous (b) and visceral adipose (c) tissues on the section 47/95 from pictures obtained by MRI. Volume of adipose tissues was determined by quantification of the voxel’s mask, from all sections of each rat, directly converted to mm^3^ by the software. MRI, Magnetic resonance imaging, Table S1: P Primers, Table S2: Gut hormones in plasma on PND105, Table S3: Gene expression in adipose tissue and hypothalamus on PND105.
