# Supplementary material for: Maternal High-Protein Diet during Pregnancy Modifies Rat Offspring Body Weight and Insulin Signalling but Not Macronutrient Preference in Adulthood
Source: Nutrients. 2019 Jan 5;11(1):96. doi: 10.3390/nu11010096 (PMC6356951; doi:10.3390/nu11010096)
Supplement: Supplementary file 1 [file nutrients-11-00096-s001.pdf]

Supplementary Materials

Methods S1. Animal procedure details during each experimental period.

General housing and care conditions:

- Animals: Wistar rats (HsdHan®:WIST, Envigo, France)
- Temperature and light: 22 + 1°C, 12h-light/12h-dark cycle with lights on at 8:00 am.
- Food weighing and turnover: Light/inactive - between 9-11:30 in the morning,
- Animal weighing: Light/inactive period - between 11:30-12:00 in the morning,
- Food and water: Free access during the entire study,
- Cages: Collective plastic cage for rat (dim in cm: 20x57x33) (Technoplast, Italy) with sawdust litter (Safe, France) (**CC**) / Individual plastic cage in rat rack (**IC**) (dim in cm: 20x42x26) (Ternox, Portugal),
- Enrichment: Polycarbonate plastic tunnel (**PPT**) (dim. L 153mm x ø 75mm),
- Diets:
  - *Standard diet:* AIN-G Normal-Protein (20% of protein) diet,
  - *Diet preparing:* Experimental diets were prepared in a specific area for animal food preparation and given as fresh food: powder reconstituted with water,
- Investigator: Same investigator took care of the animals throughout the entire experiment,
- Special ethic points: * = procedure to reduce animal number used / ** = procedure to reduce stress.

Habituation (1 week):
Six-week-old females (n=16) arrived in 3 different pools at weekly intervals and were placed in a collective cage. Six-week-old males (n=6) arrived in the first female arrival pool and were placed in a CC. Animals were placed in groups of 3 or 4 of the same gender**. Animals had free access to food (complete maintenance diet for rats, Safe, France). Cages were enriched with PPT. Animals were weighed 5 times per week.

Mating (1 week):
One female and one male were housed in a CC for 1 week without PPT to optimize mating. The experimental gestation diet was started on the first day of mating. Food was changed 3 times per week. Animals were weighed 5 times per week.

Gestation (2 weeks – D-14 to D0):
The female was separated from the male but remained in the same cage (CC). Carded cotton was added to the cage allowing the dam to construct a cotton nest. The male was returned to its original group and used again for other reproduction sessions (3 mating sessions)*. Food was changed 3 times per week. The cage was changed during weighing 10 days after male separation by transferring a part of the previous litter and cotton nest**. Female was weighed every day and considered pregnant when weight gain increased rapidly (approximately 5 grams per day at the beginning).

Birth (D0):
Birth period was approximately calculated (considering the first day of mating as the first day of gestation) to start checking every 2 hours from 8:00 am to 8:00 pm. When the dams gave birth and after approximately 4 hours, the pups were weighed. Stress was limited at this moment because dams were familiar with the investigator**. The dam was separated in another CC during pup weighing, and weighing was done as quietly as possible. Litters were normalized at 8 pups, prioritizing female pups because only females were selected for the postweaning period*. Litters were composed of 5 or 6 females and 2 or 3 males. The investigator's hands were cleaned and rubbed with the litter of the cage before each pups' litter weighing**.

Lactation (3 weeks – D0 to PND21):
Food was changed 3 times per week. The cage was changed during weighing 2 times within lactation (first time 10 days after) by transferring a part of the previous litter and cotton nest**. Dam was separated in

another CC during pup weighing, and weighing was done as quite as possible. Females and pups were weighted every day. The investigator's hands were cleaned and rubbed with the cage litter before each dam litter weighing.

Postweaning acclimation (1 week – PND21 to PND28):

Weaned selected female pups (n=2 or 3) from the same litter were placed in an IC for 4 days and were housed individually for the next 3 days, allowing a progressive separation**. Because they are very small, they can be in an IC for this first postweaning week. Females were randomly selected from the litter. The litter was selected when its growth was homogeneous. Cages were enriched with PPT. Weaned pups were weighed 5 times per week. Food was changed 3 times per week.

Postweaning diet period (11 weeks – PND28 to PND105):

Female pups randomly selected for each experimental group came from at least 3 different litters. They were housed in a new clean IC. The cage was changed every 10 days during weighing. Cages were enriched with PPT. Filter papers for feces and urine recovery were changed every day to check and weigh potential food waste. Weaned pups were weighed 5 times per week.

Sampling (PND105):

Euthanasia was performed after an overnight fasting (12 hours). Food was removed from each rat at different times to respect fasting time period.

Supplementary Materials

NPNP

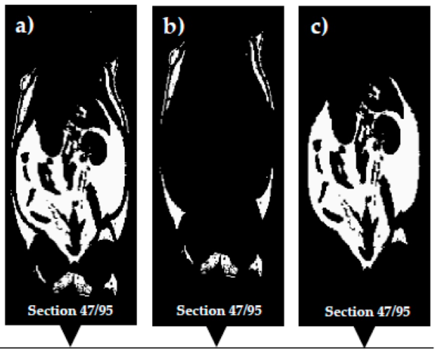

Output from quantify masks (Sections 1 to 95)	Total adipose tissue	Subcutaneous adipose tissue	Visceral adipose tissue
Number of voxels	294795	57065	237730
Volume (Millimeters^3)	41814	8094	33720

HPNP

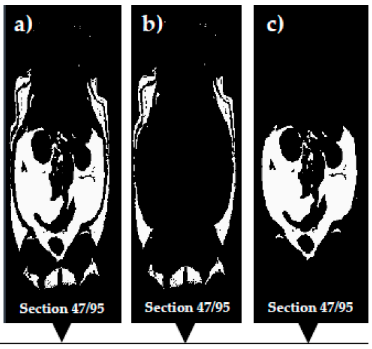

Output from quantify masks (Sections 1 to 95)	Total adipose tissue	Subcutaneous adipose tissue	Visceral adipose tissue
Number of voxels	372537	116287	256250
Volume (Millimeters^3)	52841	16494	36347

NPDSS1

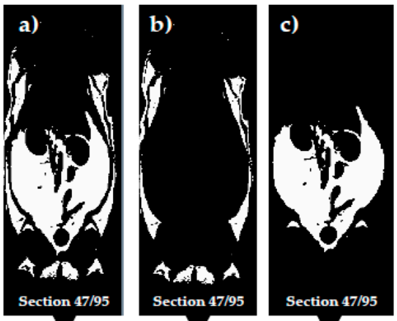

Output from quantify masks (Sections 1 to 95)	Total adipose tissue	Subcutaneous adipose tissue	Visceral adipose tissue
Number of voxels	339659	102555	237090
Volume (Millimeters^3)	48178	14547	33629

HPDSS1

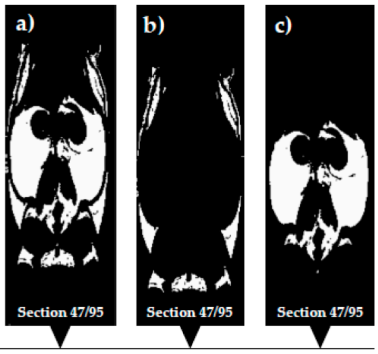

Output from quantify masks (Sections 1 to 95)	Total adipose tissue	Subcutaneous adipose tissue	Visceral adipose tissue
Number of voxels	306925	70909	236016
Volume (Millimeters^3)	43535	10058	33477

NPDSS2

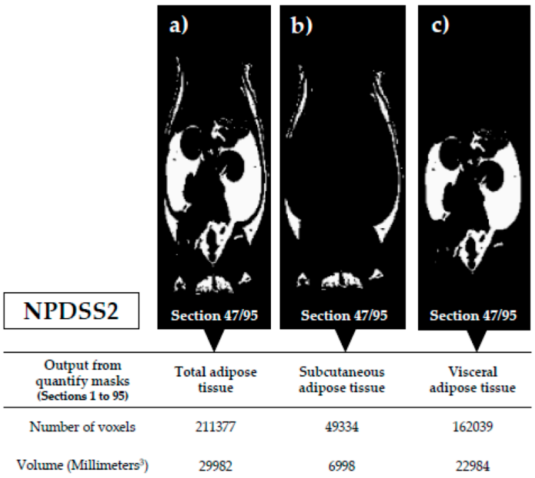

Output from quantify masks (Sections 1 to 95)	Total adipose tissue	Subcutaneous adipose tissue	Visceral adipose tissue
Number of voxels	211377	49334	162039
Volume (Millimeters^3)	29982	6998	22984

HPDSS2

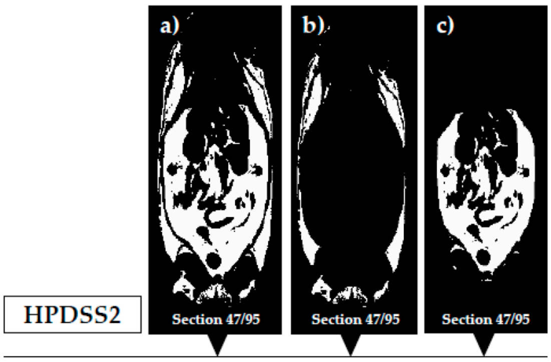

Output from quantify masks (Sections 1 to 95)	Total adipose tissue	Subcutaneous adipose tissue	Visceral adipose tissue
Number of voxels	534535	143584	390951
Volume (Millimeters^3)	75819	20366	55453

Methods F1. Example, for each diet group, of voxel masks of total (a), subcutaneous (b) and visceral (c) adipose tissues on the section 47/95 from pictures obtained by MRI. Volume of adipose tissues was determined by quantification of the voxel's mask, from all sections of each rat, directly converted to mm^3 by the software. MRI, Magnetic resonance imaging.

Supplementary Materials

Table S1. Primers

GENES	Forward primer (5′ to 3′)	Reverse Primer (3′ to 5′)	GENES	Forward primer (5′ to 3′)	Reverse Primer (3′ to 5′)
18S	ACGGAAGGGCACCACCAGGAG	GCACCACCACCCACGGAAACG	*Lepr*	TGTTCCAAACCCCAAGAATTGT	ATGCTCAAATGTTTCAGGCTTTT
Acc	TGGTGCAGAGGTACCGAAGTG	CGTAGTGGCCGTTCTGAAACT	*Lpl*	GGACTGAGGATGGCAAGCA	GGCAGGGTGAAGGGAATGTT
Agrp	TGGTGCCCTTGACCAAAGTT	AATTTCTGCCCCCACAGATG	*Npy*	GGGAGCCTGAGAAACGGC	CCTGGTGGTGGCATGCAT
Cartpt	CCGAGCCCTGGACATCTACTC	AAATACTGACCAGCTCCTTCTCATG	*Pepck*	GGAAAGTTGAATGTGTGGGTGAT	TTCTGGGTTGATGGCCCTTA
Drd1	GCCCCGAGGCTCCATCT	ACGGCATGAGGGATCAGGTA	*Pklr*	TGATGATTGGACGCTGCAA	GAGTTGGTCGAGCCTTAGTGATC
Drd2	CCATCAGCATTGACAGGTACAC	CAGTAACTCGGCGCTTGGA	*Pnpla2*	AACAGGGCTACCGAGATGGA	AGCAAAGGGTTGGGTTGGTT
Fas	TGCTCCCAGCTGCAGGC	GCCCGGTAGCTCTGGGTGTA	*Pomc*	AGGCCTTTCCCCTAGAGTTCAA	GTCGGCCTTCTCGGTATCC
Gck	TTGAGACCCGTTTCGTGTCA	AGGGTCGAAGCCCCAGAGT	*Pparg*	TCGGATCCACAAAAAGAGTAGAA	AACCTGATGGCATTGTGAGACA
Ir	TGCCCGTCTGGCTATACCAT	TCGAGGATTTGGCAGACCTT	*Scd1*	TCAGCGCTGGGAAAGTGAA	GTGTAGGAACTGGAGATCTCTTGG
Irs1	TCACAGGCAGAATGAAAGACC	TTGTGAATCGTGAAAGAGTTCGA	*Srebf1c*	GGAGCCATGCATTGCACATT	GCTTCCAGAGAGGAGGCCAG
Lep	TTCACACACGCAGTCGGTATC	CCCGGGAATGAAGTCCAAA			

ACC, Acetyl-CoA carboxylase; AGRP, Agouti-related protein; CARTPT, Cocaine- and amphetamine-regulated transcript; DRD1, Dopamine receptor 1; DRD2, Dopamine receptor 2; FAS, Fatty acid synthase; GCK, Glucokinase; IR, Insulin receptor; IRS1, Insulin receptor substrate 1; LEP, Leptin; LEPR, leptin receptor; LPL, Lipoprotein lipase; NPY, Neuropeptide Y; PEPCK, Phosphoenolpyruvate carboxykinase; PKLR, Pyruvate kinase L/R; PNPLA2, Patatin like phospholipase domain containing 2; POMC, Pro-opiomelanocortin; PPARg, Peroxisome proliferator-activated receptor gamma; SCD1, Stearoyl-CoA desaturase 1; SREBF1C, Sterol regulatory element-binding transcription factor 1 isoform c.

Supplementary Materials

Table S2. Gut hormones in plasma on PND105

**Gestation diet**		**NP**			**HP**			***P***		
**Postweaning diet**		**NP** (***n*** **= 8)**	**DSS1** (***n*** **= 8)**	**DSS2** (***n*** **= 8)**	**NP** (***n*** **= 7)**	**DSS1** (***n*** **= 8)**	**DSS2** (***n*** **= 8)**	**Gestation**	**Postweaning**	**Gestation × postweaning**
PYY	Fasted	61.7 ± 41.9	2.6 ± 0.08	2.5 ± 0.03	33.7 ± 31.2	2.5 ± 0.0	5.1 ± 2.6	0.64	0.06	0.74
(pg/mL)	fed	115.8 ± 75.6	35.3 ± 7.9	87.1 ± 21.8	94.3 ± 43.1	18.2 ± 6.4	62.4 ± 17.6	0.51	0.14	1.00
GIP	Fasted	46.1 ± 13.2	49.7 ± 9.7	44.8 ± 9.6	32.2 ± 12.5	28.9 ± 3.7	47.9 ± 12.7	0.32	0.73	0.46
(pg/mL)	fed	307.1 ± 70.3[a,b]	260.9 ± 37.0[a,b]	472.3 ± 56.2[a]	367.7 ± 74.0[a,b]	147.5 ± 16.9[b]	235.1 ± 45.4[b]	0.04 *	0.007 *	0.01 *
GLP1	Fasted	276.9 ± 200.1	80.0 ± 7.8	91.1 ± 17.0	132.6 ± 55.6	70.0 ± 0.0	98.1 ± 18.8	0.51	0.27	0.65
(pg/mL)	fed	181.5 ± 118.5	55.4 ± 12.3	38.1 ± 10.1	91.7 ± 41.5	75.9 ± 19.0	62.2 ± 23.0	0.78	0.23	0.53

Data are means ± SEMs. Effects of diets were tested within model W (*, $p < 0.05$). Means that are significantly different ($p < 0.05$) according to the post-hoc test have different letters ([a] or [b]). PYY, Peptide YY; GIP, Gastric Inhibitory Polypeptide; GLP1, Glucagon-like peptide-1; PND, Post-natal Day; NP, Normal-Protein (control); HP, High-Protein; DSS1, Dietary Self-Selection 1 (P and G/L in 2 different cups); DSS2, Dietary Self-Selection 2 (P, G and L in 3 different cups).

Supplementary Materials

Table S3. Gene expression in adipose tissue and hypothalamus on PND105

Gestation diet	NP			HP			*P*		
Postweaning diet	NP (*n* = 8)	DSS1 (*n* = 8)	DSS2 (*n* = 8)	NP (*n* = 7)	DSS1 (*n* = 8)	DSS2 (*n* = 8)	Gestation	Postweaning	Gestation × postweaning
Adipose tissue									
Fas	1.00 ± 0.46	1.16 ± 0.34	1.05 ± 0.25	1.02 ± 0.24	1.00 ± 0.17	1.72 ± 0.76	0.66	0.56	0.57
Acc	1.00 ± 0.47	1.66 ± 0.51	1.58 ± 0.33	0.99 ± 0.24	1.74 ± 0.68	1.35 ± 0.30	0.87	0.23	0.87
Scd1	1.00 ± 0.27	2.33 ± 0.78	1.61 ± 0.33	2.09 ± 0.74	2.12 ± 0.54	2.39 ± 0.84	0.37	0.53	0.50
Srebf1c	1.00 ± 0.24	0.96 ± 0.15	0.97 ± 0.19	1.35 ± 0.22	1.02 ± 0.16	1.28 ± 0.17	0.19	0.59	0.69
Lpl	1.00 ± 0.20	1.25 ± 0.15	1.26 ± 0.20	1.47 ± 0.27	1.24 ± 0.25	1.47 ± 0.27	0.33	0.89	0.55
Pnpla2	1.00 ± 0.17	1.32 ± 0.22	1.31 ± 0.23	1.49 ± 0.29	1.38 ± 0.25	1.63 ± 0.31	0.22	0.62	0.66
Lep	1.00 ± 0.27	0.89 ± 0.09	0.80 ± 0.11	1.59 ± 0.45	0.95 ± 0.08	1.45 ± 0.38	0.09	0.42	0.52
Pparg	1.00 ± 0.23	1.73 ± 0.38	1.53 ± 0.27	2.09 ± 0.39	1.98 ± 0.54	1.62 ± 0.30	0.21	0.61	0.37
Hypothalamus									
Npy	1.00 ± 0.23	1.27 ± 0.63	1.01 ± 0.26	1.66 ± 0.41	2.09 ± 1.13	1.7 ± 0.69	0.26	0.77	0.93
Agrp	1.00 ± 0.23	0.57 ± 0.14	0.54 ± 0.17	0.64 ± 0.16	0.75 ± 0.28	0.62 ± 0.15	0.75	0.23	0.37
Pomc	1.00 ± 0.25	0.87 ± 0.33	0.70 ± 0.17	1.05 ± 0.19	1.05 ± 0.28	1.10 ± 0.28	0.58	0.69	0.72
Cartpt	1.00 ± 0.21	1.30 ± 0.57	0.73 ± 0.15	1.18 ± 0.34	1.35 ± 0.46	1.30 ± 0.43	0.50	0.53	0.79
Lepr	1.00 ± 0.21	1.03 ± 0.32	0.73 ± 0.17	1.11 ± 0.21	0.98 ± 0.27	1.04 ± 0.23	0.74	0.47	0.66
Ir	1.00 ± 0.16	1.16 ± 0.37	0.90 ± 0.17	1.29 ± 0.24	0.99 ± 0.25	1.03 ± 0.26	0.76	0.60	0.56

Data are means ± SEMs. Effects of diets were tested within model W (*, $p < 0.05$). FAS, Fatty acid synthase; ACC, Acetyl-CoA carboxylase; SCD1, Stearoyl-CoA desaturase 1; SREBF1C, Sterol regulatory element-binding transcription factor 1 isoform c; LPL, Lipoprotein lipase; PNPLA2, Patatin like phospholipase domain containing 2; LEP, Leptin; PPARg, Peroxisome proliferator-activated receptor gamma; NPY, Neuropeptide Y; AGRP, Agouti-related protein; POMC, Pro-opiomelanocortin; CARTPT, Cocaine- and amphetamine-regulated transcript; LEPR, leptin receptor; IR, Insulin receptor; PND, Post-natal Day; NP, Normal-Protein (control); HP, High-Protein; DSS1, Dietary Self-Selection 1 (P and G/L in 2 different cups); DSS2, Dietary Self-Selection 2 (P, G and L in 3 different cups).
